# Isolation and Characterization of a Novel Virulent Phage ASG01 of *Aeromonas salmonicida* and Its Cell Wall Hydrolase Activity

**DOI:** 10.3390/microorganisms12030629

**Published:** 2024-03-21

**Authors:** Chen Li, Qiting Fang, Yangjun Zhang, Kunyan Li, Yaoguang Li, Rong Wang, Yuyuan Peng, Guofan Zhang, Liqiu Xia, Shengbiao Hu

**Affiliations:** 1 State Key Laboratory of Developmental Biology of Freshwater Fish, College of Life Science, Hunan Normal University, No. 36 Lushan Street, Changsha 410081, China; lichen3703@hunnu.edu.cn (C.L.); 202020141248@hunnu.edu.cn (Q.F.); 202170142773@hunnu.edu.cn (Y.Z.); 202270142917@hunnu.edu.cn (K.L.); 201730151064@hunnu.edu.cn (Y.L.); 202120142660@hunnu.edu.cn (R.W.); 202320142769@hunnu.edu.cn (Y.P.); zgf@hunnu.edu.cn (G.Z.); xialq@hunnu.edu.cn (L.X.); 2 Hunan Provincial Key Laboratory of Microbial Molecular Biology, College of Life Science, Hunan Normal University, No. 36 Lushan Street, Changsha 410081, China

**Keywords:** aquaculture, antibacterial agent, heterologous expression, endolysin, site-directed mutation

## Abstract

*Aeromonas salmonicida* is an important pathogen that causes furunculosis in trout and salmon with high morbidity and mortality, resulting in significant economic losses in aquaculture. Overuse of antibiotics has led to the continuous emergence of drug-resistant strains. Hence, there is an urgent need to find an alternative environmentally friendly antimicrobial agent. In this study, we isolated a virulent phage of *A. salmonicida*, named ASG01, which belongs to the Myoviridae family and maintains lytic activity at a pH value range from 4 to 12 and in the temperature range from 30 °C to 60 °C. The whole genomic sequence of ASG01 showed 82% similarity to *Aeromonas* phage pAh6-C. The cell wall hydrolase (Cwh)-encoding gene from the genome of ASG01 was predicted and heterologously expressed. Notably, in the absence of additional phage genes, endogenous expression of Cwh could lyse *E. coli* cells and greatly inhibit the growth of tested fish pathogenic bacteria. The lytic activity of Cwh was eliminated when the predicted active site was mutated. These results indicate that Cwh of ASG01 possessed excellent lytic activity and a wide antibacterial spectrum, suggesting its potential as an effective enzybiotic.

## 1. Introduction

The Gram-negative bacterium *Aeromonas salmonicida*, which is the only non-motile species in the genus *Aeromonas* [1], is responsible for typical furunculosis and bacterial septicemia and has been considered as one of the most important pathogens in a variety of fish species in aquaculture [2,3,4,5], causing significant economic losses in aquaculture [6]. Recently, *A. salmonicida* was isolated from the human blood in a case report [7]. Clinical control of *A. salmonicida* in worldwide aquaculture still mainly relies on commercialized antibiotics [8]. However, the abuse of antibiotics has led to the recent emergence of antibiotic-resistant *A. salmonicida* strains in aquaculture, which poses a risk to environment and human health [9]. Therefore, there is an urgent need to develop alternative measures to treat furunculosis caused by *A. salmonicida.*

Bacteriophages (phages) are bacterial viruses that can specifically infect host bacterial cells, disrupt bacterial metabolism, and lyse host cells [10]. Phage therapy, which is based on the use of phages to reduce or eliminate pathogenic bacteria, has been proven to be a promising and flexible approach to combat bacterial infections in aquaculture [11]. Remarkably, several studies have shown that phage treatment can significantly reduce or even prevent the death of fish infected with *A. salmonicides* and have generally demonstrated the potential efficacy of phages against *A. salmonicida* infections [3,12,13].

At the end of the phage lytic life cycle, a lytic cassette will be produced to lyse the host cell and release the newly produced virions [14]. Endolysin, a component of the lytic cassette, is responsible for cleaving peptidoglycan (PG) of target bacteria, the main component of the bacterial cell wall to protect cells from expansion and rupture [15,16]. The structure of endolysins can be either globular or modular [17]. Generally, the endolysins from phages that infect Gram-negative bacteria are single-domain globular proteins typically composed of an enzymatically active domain (EAD) which can catalyze the hydrolysis of PG layer [18]. In contrast, endolysins from Gram-positive-infecting phages typically utilize a modular design, consisting of multiple domains with separate activities [19]. In addition to one or more EADs, they also comprise cell wall-binding domains (CBDs) that recognize epitopes on the surface of susceptible hosts [20].

Since 2001, scientists have begun exploring the utility of endolysins in animal infection models of human disease [15]. In Gram-positive bacteria, when added externally, small quantities of purified recombinant endolysins can cause immediate lysis of the target bacterium [21,22]. However, in Gram-negative bacteria, the outer membrane which serve as a natural barrier restricting endolysin’s access to peptidoglycan, hindering the extracellular use of endolysins as anti-Gram-negative bacteria agents [23,24]. Recently, several chimeric endolysins in which the *N*-terminal of endolysin was fused with a short peptide with membrane-penetrating capabilities have been constructed and used to kill Gram-negative bacteria, opening up new possibilities to increase their antibacterial potential and broaden the spectrum of their applications. Artilysins, which were produced by combining a polycationic nonapeptide (PCNP; a mixture of arginine and lysine residues) and a modular endolysin, exhibited high antibacterial activity against multidrug-resistant *Pseudomonas aeruginosa* strains [25]. The artilysin Art-175 represents another class of artilysins, based on the fusion of an antimicrobial peptide (AMP) and an endolysin. Art-175 inhibited all *P. aeruginosa* strains tested, irrespective of a clinical or environmental origin and irrespective of the degree of multi-antibiotic resistance [26].

In the current study, we isolated and characterized a virulent phage (designated as ASG01) that could infect *A. salmonicida* in the aquatic environment. We described the biological properties and complete genome sequence of the ASG01 phage. By analyzing the genomic sequence of ASG01, its putative encoding sequence of endolysin (designated as LysASG01) was cloned and expressed. The conserved residues in the vicinity of the active site of ASG01Lys were analyzed by using point mutation.

## 2. Materials and Methods

### 2.1. Bacterial Strains, Plasmids and Culture Conditions

*E. coli* DH5α and Rosetta (pLys) strains were used for DNA cloning and recombinant endolysin expression, respectively. Plasmid pET28a was used for recombinant endolysin expression in the Rosetta (pLys) strain. Bacteria and phages were cultured in Luria–Bertani (LB) broth at 30 °C with shaking, except for *A. salmonicida* JF2267 (20 °C). Isopropyl-beta-D-thiogalactoside (IPTG) (Coolaber, Beijing, China) was used as an inducer at a concentration of 0.5 mM.

### 2.2. Phage Isolation and Enrichment

Sewage samples were collected from Xiangjiang River and clarified by centrifugation at 4000 rpm for 15 min followed by filtration through a 0.22 μm filter (Biosharp, Hefei, China). Soft agar was used in a 100 μL fresh overnight culture of *A. salmonicida* DBFF01 and 10 μL of filtrate was spotted and cultured a 30 °C overnight. The existent of phages was demonstrated by the presence of a clear zone on the plate. Finally, phage purification was performed using a modified double-layer AGAR plate method [27]. Briefly, the cleared bacteria–phage co-culture was centrifuged at 9000× *g* for 15 min at 4 °C, and bacteria were removed by filtration. The top agar layer (0.7% agar) containing the filtrate mixed with fresh host culture was poured onto the bottom agar (1.8%). The host range was tested with the same method. The information regarding the tested strains is shown in Table 1.

### 2.3. Phage Concentration

A volume of 500 mL of phage lysate was centrifuged at 9500 rpm for 30 min, and 100 μL DNase A (10 mg/ML) (Takara Biomedical Technology, Beijjing, China) and 50 μL RNase I (20 mg/ML) (Takara Biomedical Technology, Beijjing, China) were added to the supernatant and incubated for 1 h at 37 °C. Then, 1 M NaCl (Shhushi, Shanghai, China) was added and incubated for 1 h at 4 °C. The preparation was centrifuged at 9500 rpm for 30 min and phages were precipitated using 0.1 M PEG8000 (Biotopped, Beijing, China) overnight at 4 °C. Phages were harvested by means of centrifugation at 9500 rpm for 30 min and the sediment was resuspended in 2 mL of LB. Finally, phages were purified with chloroform (Shhushi, Shanghai, China) (phage:chloroform = 1:1). Phages were harvested from the supernatant and stored in 30% glycerol (Shhushi, Shanghai, China) at 4 °C [28].

### 2.4. Transmission Electron Microscopy

A 15 μL phage suspension was applied on a copper mesh with a supporting membrane and negatively stained with 2% phosphotungstic acid (Shhushi, Shanghai, China). Specimens were observed using a transmission electron microscope (Hitachi HT7700, Tokyo, Japan) at an acceleration voltage of 80 kV.

### 2.5. Phage Physiological Characteristics Quantification

The pH dependence of the ASG01 was assayed by mixing the phage filtrate and LB with different pH values within the range of 1–14. A double-layer agar plate was used to assess the activity of phages. Thermostability was evaluated by incubating phages at several temperatures (30 °C, 40 °C, 50 °C, 60 °C, 70 °C, 80 °C) for 1 h, and 100 μL was used for the agar plate assay.

### 2.6. Multiplicity of Infection (MOI)

MOI refers to the ratio of phages to host bacteria at the beginning of an infection. Equal volumes of phage solution and host bacterial solution were mixed in sterile LB at MOI (0.0001 to 100). The mixture was incubated for 4 h at 30 °C. Next, free phages were removed by means of centrifugation, the phage titer was determined, and the infection complex with the highest titer was selected as the best. All experiments were performed in triplicate.

### 2.7. Phage DNA Extraction, Sequencing and Bioinformatic Analysis

Phage DNA was extracted using the phage DNA isolation kit (Abigen, Beijing, China) and genomes were sequenced by GENEWIZ (GENEWIZ, Suzhou, China). The whole genomes were sequenced with Illumina (San Diego, CA, USA) MiSeq 2500 and assembled with SOAPdenovo v2.01 [29]. The tool ORF Finder (https://www.ncbi.nlm.nih.gov/orffinder/, accessed on 20 December 2019) was used to predict open reading frames (ORFs), and a BLASTP (https://blast.ncbi.nlm.nih.gov/Blast.cgi, accessed on 20 December 2019) analysis was performed to assign functional annotations to the predicted ORFs. The CG view was used to create genome maps [30]. Phylogenetic analysis of the phages and proteins were performed by using MEGA6 with the neighbor-joining method and an alignment with Clustal implemented in MEGA [31].

### 2.8. Bioinformatics Analysis of Cell Wall Hydrilase (Cwh) Protein

National Center for Biotechnology Information (NCBI) Conserved Domains (https://www.ncbi.nlm.nih.gov/Structure/cdd/wrpsb.cgi, accessed on 2 July 2020) worked to analyze the conservative structure domain. ProtParam online tools (https://web.expasy.org/protparam/, accessed on 15 April 2020) worked to analyze the physical and chemical properties of proteins. TMHMM 2.0 (http://www.cbs.dtu.dk/services/TMHMM/, accessed on 15 April 2020) predicted the transmembrane protein structure domain. SignalP5.0 (http://www.cbs.dtu.dk/services/SignalP/, accessed on 15 April 2019) worked to analyze its signal peptide. Protscale (https://web.expasy.org/protscale/ accessed on 15 April 2019) predicted the hydrophilic/hydrophobic protein. SOPMA (https://npsa-prabi.ibcp.fr/cgi-bin/npsa_automat.pl?page=npsa_sopma.html, accessed on 15 April 2019) predicted its secondary structure. The Swiss-Model online tools (https://swissmodel.expasy.org/, accessed on 18 April 2019) were used for protein tertiary structure prediction.

### 2.9. Construction of Engineered Strains

All variants of *cwh* for *E. coli* were cloned into the vector of pET28a, which allowed the expression of target genes to be induced under the control of the *lac* promotor using IPTG as the inducer. For higher expression in host bacteria, the *cwh* gene was synthesized after codon optimization at Sangon (Sangon Biotech, Shanghai, China). The sequence and pET28a plasmid were digested with *Sal* I (New England Biolabs, Ipswich, MA, USA) and *Bam*H I (New England Biolabs, MA, USA) at 37 °C for 4 h, and subsequently the gel was purified. The digested vector and target gene were ligated using T4 DNA ligase (Takara Biomedical Technology, Beijjing, China) at 16 °C overnight. The ligation solution was transformed into *E. coli* DH5α to construct the recombinant plasmid pET28a-*cwh*. Then, it was transformed into *E. coli* Rosetta (pLys). The site-directed mutagenesis was performed on *cwh*, and the site-directed mutagenesis kit was used according to the manufacturer’s protocol (Beyotime Biotechnology, Shanghai, China). The pBAD promoter and araC regulatory protein gene were added in the front of the *cwh* gene sequence to regulate its expression. The sequence was synthesized by Sangon Biotech and inserted into the vector pEVS107 to construct the recombinant plasmid pEVS107-pBAD-*cwh*. The plasmid then was transformed into *A. salmonicida* DBFF01, *A. salraonicida* JF2267 and *A. hydrophila* by means of conjugational transfer. In order to induce the expression of the Cwh, 10% L-arabinose (BBI, Shanghai, China) was used.

### 2.10. Lytic Activity Assays of Cwh

Twenty milliliter cultures were induced for 3.5 h and OD_600_ was measured before and after induction to evaluate the cell viability. One hundred milliliter cultures were diluted in ddH_2_O and plated on LB agar plates to perform spot dilution assays. Colonies were counted after overnight culture.

### 2.11. Scanning Electron Microscopy

The lysate was centrifuged at 8000 rpm for 5 min and the bacteria were washed 5 times with LB. A fixative (600 µL of 2.5% glutaraldehyde) (Shhushi, Shanghai, China) was added to fix lucifugally overnight at 4 °C. The cell was dehydrated with 30%, 50%, 70%, 90%, and 95% absolute ethanol (Shhushi, Shanghai, China), (1 min each), and finally resuspended in 95% absolute ethanol. Specimens were observed using a scanning electron microscope (Hitachi SU8010, Tokyo, Japan) at an acceleration voltage of 2.0 kV.

### 2.12. Statistical Analysis

Statistical analysis of bar chart was performed using GraphPad Prism v8.0.2 (GraphPad Software, Boston, WA, USA). Data are represented as mean ± SEM, and ns (no significance) indicates *p* > 0.05; * indicates *p* < 0.05; ** indicates *p* < 0.01; and *** indicates *p* < 0.001.

## 3. Results

### 3.1. Phage Isolation and Basic Characterization

**Figure 1:**
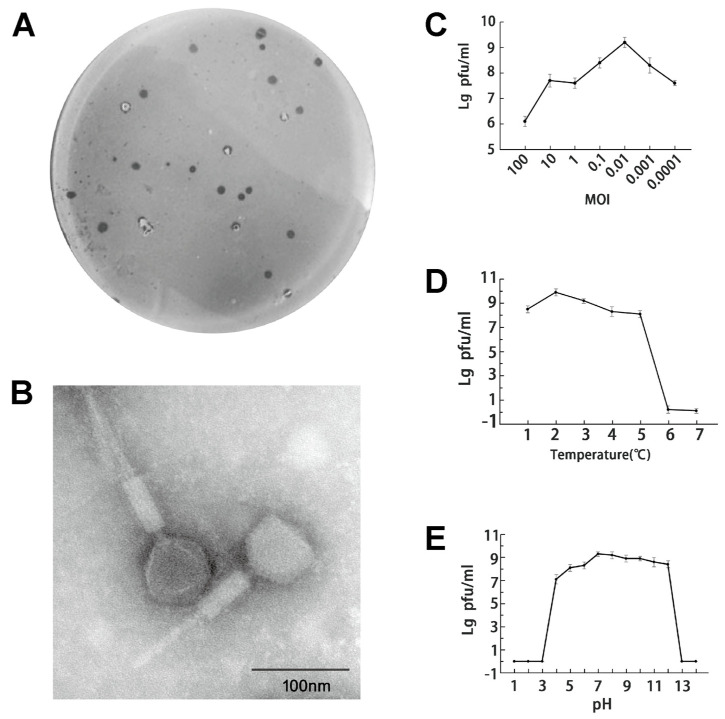
Isolation, morphology and biological properties of ASG01. (**A**) Plaques formed on *Aeromonas salmonicida* DBFF01 agar plate. (**B**) Transmission electron micrographs reveal the morphology of phages. Scale bar = 100 nm. (**C**) Determination of optimal multiplicity of infection (MOI) of phage of ASG01. (**D**) Stability analysis of ASG01 temperature (30–80 °C). (**E**) Stability analysis of ASG01 pH (1–14).

ASG01 formed clear plaques in spots (Figure 1A). When observed in transmission electron microscopy (TEM) images, the phage ASG01 had a ~24 nm icosahedral head with a ~157 nm flexible tail (Figure 1B) and was classified into the family Myoviridae. Host range analysis showed that ASG01 had a narrow host spectrum and could only specifically infect its host bacterium DBFF01, while it had no lytic effect on other tested pathogens (Table 1). Usually, a narrow host range can reduce the damage to other probiotics, increasing its potential as a biological agent. At a MOI of 0.01, the phage titer reached the highest value of ~10^9^ PFU/mL (Figure 1C), indicating that the optimal MOI of bacteriophage ASG01 was 0.01. Its lytic activity against DBFF01 was still stable in the temperature range from 30 °C to 60 °C, but was completely lost at 70 °C (Figure 1D). ASG01 possesses highly lytic activity at pH values ranging from 4.0 to 12.0. When the pH was lower than 3.0 or higher than 13.0, its lytic activity was completely lost (Figure 1E).

### 3.2. Genome Analysis of ASG01

**Figure 2:**
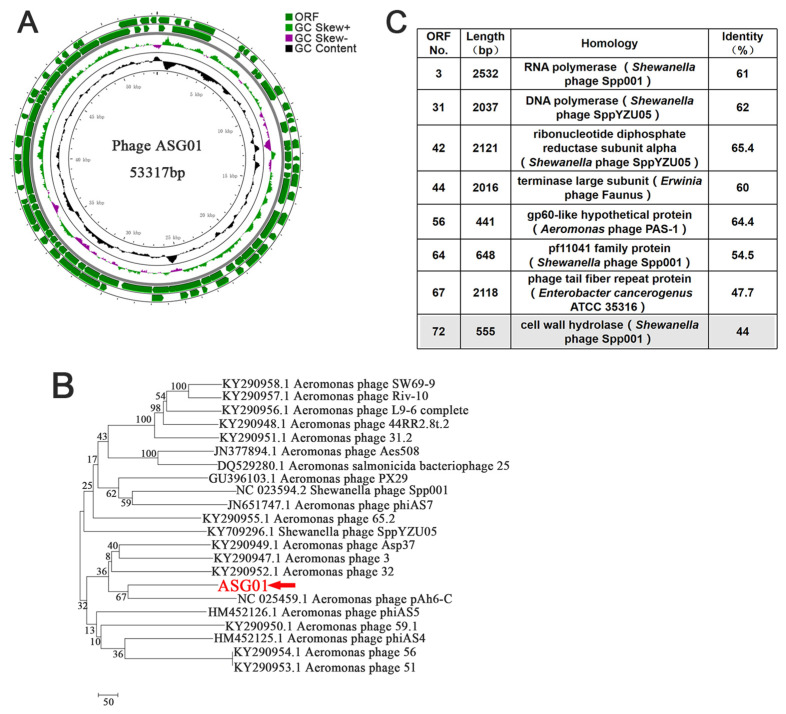
Genome sequencing analysis of the phage ASG01. (**A**) Graphical representation of the phage ASG01. Circles show (from the outside to the inside) (1) the coding seqs counter-clockwise; (2) the coding seqs clockwise; (3) GC skew. Values greater than zero are in green, while those lower than zero are in purple; (4) G + C% content; (5) physical map scaled in kb. (**B**) Neighbor-joining phylogenetic tree based on the complete genome sequence of phage ASG01. (**C**) The genome annotation of ASG01.

The genome of ASG01 is a circular double-stranded DNA molecule, which consists of 53,317 bp with a total GC content of 51.79% (Figure 2A). The ASG01 genome showed 82% identity with the *Aeromonas* bacteriophage pAh6-C, which was consistent with the resulting phylogenetic cluster based on the entire genome (Figure 2B), representing a new phage species. Seventy-three open reading frames (ORFs) were predicted in the ASG01 genome. Eight functionally annotated proteins were predicted and could be categorized into four groups (Figure 2C), namely (1) DNA replication and regulation proteins (RNA polymerase and DNA polymerase); (2) structural proteins (tail fiber repeat protein); (3) packaging proteins (the large subunit of the terminal enzyme, which was reported to possess multiple activities of ATPase, DNA ligase and restriction endonuclease [32]; and (4) lysis proteins (cell wall hydrolase). Besides Cwh, many virulent phages also contain accessory proteins, such as holin, pinholin or spanins [33,34,35]). However, these accessory proteins were absent from the genome of ASG01.

### 3.3. ASG01 Lysis Protein and Its Bioinformatic Analysis

**Figure 3:**
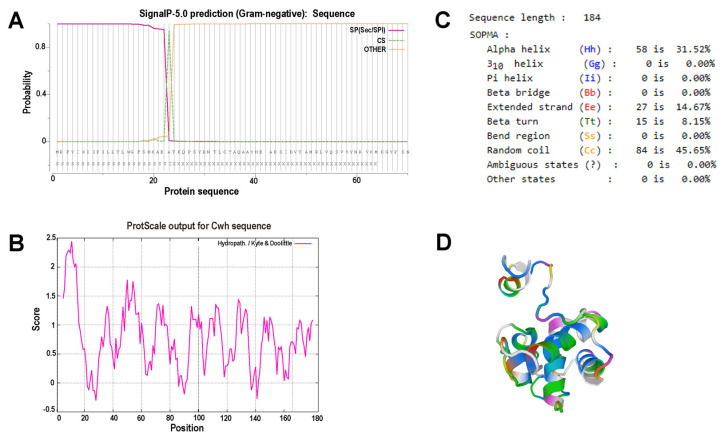
Bioinformatic analysis of Cwh protein. (**A**) Signal peptide prediction. (**B**) Hydrophili-hydrophobic analysis. (**C**) Secondary structure prediction. (**D**) Tertiary structure prediction.

As ORF72 was predicted to encode Cwh, we analyzed its molecular characteristics by using bioinformatic tools. TMHMM analysis showed that it did not have a transmembrane domain and a signal peptide was annotated in the *N*-terminal by using SignalP5.0 (Figure 3A). Cwh has far more hydrophobic residues than hydrophilic residues (Figure 3B). SOPMA analysis of the secondary structure results showed that α-helixes accounted for 31.52%, extended strands accounted for 14.67%, β-turns accounted for 8.15%, and random coils accounted for 45.65% of the structure (Figure 3C). The 3D structure was also predicted by using Swiss-Model tools (https://swissmodel.expasy.org/, accessed on 18 April 2019) (Figure 3D).

### 3.4. Lytic Activity Analysis of Cwh in E. coli

**Figure 4:**
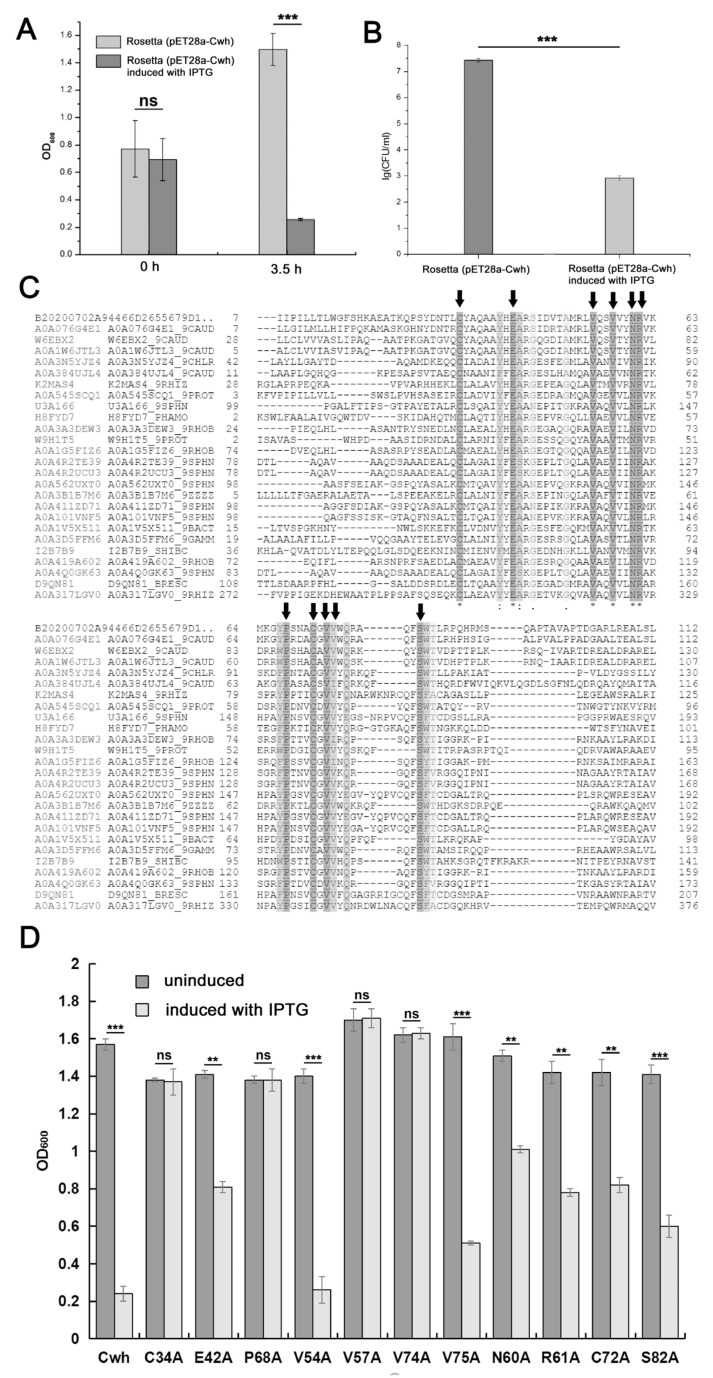
Analysis of antimicrobial activity and key active sites of Cwh. (**A**) Antimicrobial activity of Cwh by OD_600_, (**B**) analysis of antimicrobial activity of Cwh by means of cell counting in Rosetta (pLys), (**C**) conserved amino acid residue analysis based on Uniprot data alignment, the arrows represent the eleven conserved amino acids in Cwh, and (**D**) lysis activity assay of the mutant protein in Cwh protein. Statistical analysis was performed with GraphPad (**, *p* < 0.01; ***, *p* <  0.001, ns, not significant).

The Cwh-encoding gene was then cloned into pET28a and heterologously expressed in *E. coli* Rosetta (pLys). After being induced by IPTG for 3.5 h, the OD_600_ value of *E. coli* was dramatically decreased (Figure 4A) and the number of *E. coli* cells decreased by 10^5^ CFU (Figure 4B). This indicates that the lytic activity of Cwh is independent of accessory proteins (holin, pinholin or spanins). As the rapid cell lysis occurred upon induction, we failed to purify Cwh and cannot accurately assess its lytic activity. The presence of *N*-terminal signal peptides suggested that Cwh might be secreted through the Sec secretion pathway [36]. Cwh contains a hydrolase-2 domain, which was first reported to hydrolyze cell wall peptidoglycan in *Bacillus subtilis* [37].

### 3.5. Key Active Sites of Cwh Protein

In order to further explore the active sites of the Cwh protein, we aligned the amino acid sequences of Cwh proteins from 26 virulent phages, and 11 highly conservative amino acid residues (E_42_, C_34_, P_68_, V_54_, V_57_, V_74_, V_75_, N_60_, R_61_, C_72_, and S_82_) were found (Figure 4C). To explore the contribution of these conservative amino acid residues to the lytic activity of Cwh, we mutated these amino acid residues into alanine, respectively. The lytic activity of C_34_A, P_68_A, V_57_A and V_74_A were completely lost, indicating that these four amino acid residues played crucial roles in the process of bacterial cell wall cleavage (Figure 4D). Bioinformatic analysis demonstrated that P_68_, V_57_ and V_74_ were located in the lysis active domain, while C_34_ participated in the formation of disulfide bonds. The lytic activity of E_42_A, N_60_A, R_61_A, C_72_A, V_75_A and S_82_A also showed a significant decrease, although it did not completely disappear, suggesting that these six amino acid residues were necessary for the full activity. The only exception is V_54_A, which possessed a similar level of lytic activity to the wild-type Cwh (Figure 4D).

### 3.6. Heterologous Expression of Cwh in Fish Pathogenic Bacteria

**Figure 5:**
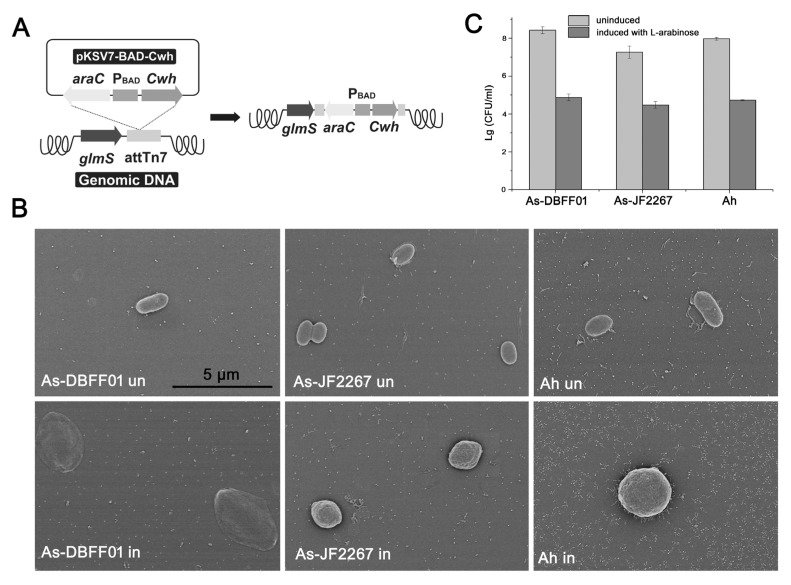
Analysis of antimicrobial activity of Cwh in pathogen (As, *Aeromonas salmonicida*; Ah, *Aeromonas hydrophila*). (**A**) Construction of the engineered strain. (**B**) Scanning electron microscope observation of cells; un, untreated; in, Cwh expressed. (**C**) Antimicrobial activity of Cwh by means of cell counting in pathogens.

The encoding gene of Cwh was cloned and placed under the control of the pBAD promoter which could by induced by L-arabinose (Figure 5A) and introduced into *A. salmonicida* DBFF01, *A. salraonicida* JF2267 and *A. hydrophila* [38]. After being induced by L-arabinose, the morphology of bacterial cells became round, swollen, and prone to rupture (Figure 5B) and the number of live bacteria of all three strains decreased by 103 (Figure 5C). These results reveal that Cwh could penetrate the membrane to hydrolyze peptidoglycan when expressed in pathogens.

## 4. Discussion

A novel phage, ASG01, isolated using *A. salmonicida* DBFF01 as the indicator strain, showed a different lytic pattern and host spectrum from common virulent phages. ASG01 belongs to the Myoviridae family and maintains lytic activity over a wide range of temperatures and pH values. This phage only infects its host strain DBFF01, showing an extremely narrow lytic spectrum. It is considered that a broad bactericidal spectrum against pathogens is essential for therapeutic candidates [39]. However, lysin with a variety of bactericidal activities may also affect the healthy microbiome in fish intestines. Therefore, compared with other broad-spectrum phages, ASG01 has greater potential to control infections caused by *A. salmonicida* without excessively interfering with other symbiotic microorganisms.

In recent years, there are many reports on the bactericidal effect of cell wall hydrolase [22,40], but there are few focused on fish pathogens. Moreover, the successful application of endolysins would be impossible without a comprehensive understanding of their biochemical, biophysical and antimicrobial properties [41]. In this study, we identified and characterized Cwh, a novel endolysin that can lead to cell lysis when expressed in vivo, which may be related to its unusual domain. The N-terminal of Cwh contains a signal peptide of 23 amino acids, which may play a role in transport outside the cell membrane via the general secretory pathway [42]. Moreover, the Cwh protein contains a hydrolase-2 domain, and site-directed mutagenesis experiments showed that the hydrolase-2 domain and disulfide bonds contribute to cell lysis. This finding enhances our understanding of the mechanism of host cell lysis. At the same time, since this study provides indirect evidence for the potential role of signal peptides in Cwh function, it is necessary to further investigate their specific role in cells.

We found that Cwh protein has no lysis effect on Gram-negative bacteria in vitro, which may be due to the presence of an outer membrane layer of the cell wall in Gram-negative bacteria [43]. Fortunately, other strategies have been proven to effectively enhance the hydrolysis of Gram-negative bacteria by peptidoglycan hydrolase, such as fusing cell wall hydrolase with short peptides that can penetrate the membrane to kill bacteria in vitro [44]. In addition, there are many strategies to improve the lytic activity of cell wall hydrolases, such as combining cell wall-binding domains with existing cell wall hydrolases to increase the affinity of hydrolases to the cell wall [45]. All in all, through the study of its lytic activity, the Cwh protein is expected to be designed as a more effective antibacterial agent.

## 5. Conclusions

In this study, we isolated a virulent phage of *A. salmonicida*, named ASG01, which belongs to the Myoviridae family and maintains high activity at a pH of 4 to 12 and a temperature of 30 °C to 60 °C, respectively. Notably, in the absence of additional phage genes, endogenous expression of Cwh can lyse *E. coli* cells. In addition, the expression of Cwh inhibited the growth of tested fish pathogenic bacteria. The lytic activity of Cwh was eliminated when the predicted key active site was mutated. These results indicate that Cwh of ASG01 possesses excellent lytic activity and a wide antibacterial spectrum, suggesting its potential as an effective enzybiotic.

## Figures and Tables

**Table 1:** The detection of host range of phage ASG01.

Bacterial Strain	Spot of ASG01 ^a^	Strain Resource ^b^
*Aeromonas salmonicida* DBFF01	+	HNNU
*Aeromonas salmonicida* JF2267	−	UBIVB
*Plesiomonas shigelloides*	−	HNNU
*Aeromonas veronii*	−	HNNU
*Aeromonas hydrophila*	−	HNNU
*Aeromonas sobria*	−	HNNU
*Aeromonas jandaei*	−	HNNU
*Escherichia coli*	−	HNNU

^a^ + observed; − were not observed; ^b^ HNNU, Hunan Normal University; UBIVB, University of Bern Institute of Veterinary Bacteriology.

## Data Availability

The raw data supporting the conclusions of this article will be made available by the authors on request.

## References

[1] Park S.Y., Han J.E., Kwon H., Park S.C., Kim J.H. (2020). Recent Insights into *Aeromonas salmonicida* and its bacteriophages in aquaculture: A Comprehensive Review. J. Microbiol. Biotechnol..

[2] Valderrama K., Soto-Dávila M., Segovia C., Vásquez I., Dang M., Santander J. (2019). *Aeromonas salmonicida* infects atlantic salmon (*Salmo salar*) erythrocytes. J. Fish Dis..

[3] Imbeault S., Parent S., Lagacé M., Uhland C.F., Blais J. (2006). Using bacteriophages to prevent furunculosis caused by *Aeromonas salmonicida* in farmed brook trout. J. Aquat. Anim. Health.

[4] Lian Z., Bai J., Hu X., Lü A., Sun J., Guo Y., Song Y. (2020). Detection and characterization of *Aeromonas salmonicida* Subsp. *salmonicida* infection in crucian carp carassius auratus. Vet. Res. Commun..

[5] Lago E., Nieto T., Farto R. (2012). irulence Factors of *Aeromonas salmonicida* Subsp. *salmonicida* strains associated with infections in turbot psetta maxima. Dis. Aquat. Org..

[6] Chukwu-Osazuwa J., Cao T., Vasquez I., Gnanagobal H., Hossain A., Machimbirike V.I., Santander J. (2022). Comparative reverse vaccinology of *Piscirickettsia salmonis*, *Aeromonas salmonicida*, *Yersinia ruckeri*, *Vibrio anguillarum* and *Moritella viscosa*, frequent pathogens of atlantic salmon and Lumpfish aquaculture. Vaccines.

[7] Tewari R., Dudeja M., Nandy S., Das A.K. (2014). Isolation of *Aeromonas salmonicida* from human blood sample: A Case Report. J. Clin. Diagn. Res..

[8] Desbois A.P., Cook K.J., Buba E. (2020). Antibiotics modulate biofilm formation in fish pathogenic isolates of atypical *Aeromonas salmonicida*. J. Fish Dis..

[9] Tanaka K.H., Vincent A.T., Trudel M.V., Paquet V.E., Frenette M., Charette S.J. (2016). The mosaic architecture of *Aeromonas salmonicida* Subsp. *salmonicida* pAsa4 plasmid and its consequences on antibiotic resistance. PeerJ.

[10] Abedon S.T., Kuhl S.J., Blasdel B.G., Kutter E.M. (2011). Phage treatment of human infections. Bacteriophage.

[11] Ramos-Vivas J., Superio J., Galindo-Villegas J., Acosta F. (2021). Phage therapy as a focused management strategy in aquaculture. Int. J. Mol. Sci..

[12] Kim J.H., Choresca C.H., Shin S.P., Han J.E., Jun J.W., Park S.C. (2015). Biological control of *Aeromonas salmonicida* Subsp. *salmonicida* infection in rainbow trout (*Oncorhynchus Mykiss*) using *Aeromonas* phage PAS-1. Transbound. Emerg. Dis..

[13] Silva Y.J., Moreirinha C., Pereira C., Costa L., Rocha R.J.M., Cunha Â., Gomes N.C.M., Calado R., Almeida A. (2016). Biological control of aeromonas salmonicida infection in juvenile Senegalese sole (Solea senegalensis) with phage AS-A. Aquaculture.

[14] Obeso J.M., Martínez B., Rodríguez A., García P. (2008). Lytic activity of the recombinant staphylococcal bacteriophage ΦH5 endolysin active against *Staphylococcus Aureus* in Milk. Int. J. Food Microbiol..

[15] Nelson D., Loomis L., Fischetti V.A. (2001). Prevention and elimination of upper respiratory colonization of mice by group a *Streptococci* by using a bacteriophage lytic enzyme. Proc. Natl. Acad. Sci. USA.

[16] Attai H., Rimbey J., Smith G.P., Brown P.J.B. (2017). Expression of a peptidoglycan hydrolase from lytic bacteriophages Atu_ph02 and Atu_ph03 triggers lysis of agrobacterium tumefaciens. Appl. Environ. Microbiol..

[17] Oliveira H., Melo L.D.R., Santos S.B., Nóbrega F.L., Ferreira E.C., Cerca N., Azeredo J., Kluskens L.D. (2013). Molecular aspects and comparative genomics of bacteriophage endolysins. J. Virol..

[18] Oliveira H., Vilas Boas D., Mesnage S., Kluskens L.D., Lavigne R., Sillankorva S., Secundo F., Azeredo J. (2016). Structural and enzymatic characterization of ABgp46, a novel phage endolysin with broad anti-gram-negative bacterial activity. Front. Microbiol..

[19] Son B., Kong M., Cha Y., Bai J., Ryu S. (2020). Simultaneous control of *Staphylococcus aureus* and *Bacillus cereus* using a hybrid endolysin LysB4EAD-LysSA11. Antibiotics.

[20] Díez-Martínez R., De Paz H.D., García-Fernández E., Bustamante N., Euler C.W., Fischetti V.A., Menendez M., García P. (2015). A novel chimeric phage lysin with high in Vitro and in Vivo bactericidal activity against *Streptococcus pneumoniae*. J. Antimicrob. Chemother..

[21] Fischetti V.A. (2008). Bacteriophage lysins as effective antibacterials. Curr. Opin. Microbiol..

[22] Schuch R., Lee H.M., Schneider B.C., Sauve K.L., Law C., Khan B.K., Rotolo J.A., Horiuchi Y., Couto D.E., Raz A. (2014). Combination therapy with lysin CF-301 and antibiotic is superior to antibiotic alone for treating methicillin-resistant *Staphylococcus aureus*–induced murine bacteremia. J. Infect. Dis..

[23] Gutiérrez D., Briers Y. (2021). Lysins breaking down the walls of gram-negative bacteria, no longer a no-go. Curr. Opin. Biotechnol..

[24] Zampara A., Sørensen M.C.H., Gencay Y.E., Grimon D., Kristiansen S.H., Jørgensen L.S., Kristensen J.R., Briers Y., Elsser-Gravesen A., Brøndsted L. (2021). Developing innolysins against *Campylobacter jejuni* using a novel prophage receptor-binding protein. Front. Microbiol..

[25] Briers Y., Walmagh M., Van Puyenbroeck V., Cornelissen A., Cenens W., Aertsen A., Oliveira H., Azeredo J., Verween G., Pirnay J.-P. (2014). Engineered endolysin-based “Artilysins” to combat multidrug-resistant gram-negative pathogens. mBio.

[26] Defraine V., Schuermans J., Grymonprez B., Govers S.K., Aertsen A., Fauvart M., Michiels J., Lavigne R., Briers Y. (2016). Efficacy of Artilysin Art-175 against resistant and persistent *Acinetobacter baumannii*. Antimicrob. Agents Chemother..

[27] Santos S.B., Carvalho C.M., Sillankorva S., Nicolau A., Ferreira E.C., Azeredo J. (2009). The use of antibiotics to improve phage detection and enumeration by the double-layer agar technique. BMC Microbiol..

[28] Yamamoto K.R., Alberts B.M., Benzinger R., Lawhorne L., Treiber G. (1970). Rapid bacteriophage sedimentation in the presence of polyethylene glycol and its application to large-scale virus purification. Virology.

[29] Luo R., Liu B., Xie Y., Li Z., Huang W., Yuan J., He G., Chen Y., Pan Q., Liu Y. (2012). SOAPdenovo2: An empirically improved memory-efficient short-read de novo assembler. GigaScience.

[30] Stothard P., Wishart D.S. (2005). Circular genome visualization and exploration using CGView. Bioinformatics.

[31] Tamura K., Stecher G., Peterson D., Filipski A., Kumar S. (2013). MEGA6: Molecular Evolutionary Genetics Analysis Version 6.0. Mol. Biol. Evol..

[32] Oram M., Sabanayagam C., Black L.W. (2008). Modulation of the packaging reaction of bacteriophage T4 terminase by DNA structure. J. Mol. Biol..

[33] White R., Chiba S., Pang T., Dewey J.S., Savva C.G., Holzenburg A., Pogliano K., Young R. (2011). Holin triggering in real time. Proc. Natl. Acad. Sci. USA.

[34] Young R. (2013). Phage lysis: Do we have the hole story yet?. Curr. Opin. Microbiol..

[35] Young R. (2014). Phage lysis: Three steps, three choices, one outcome. J. Microbiol..

[36] São-José C., Parreira R., Vieira G., Santos M.A. (2000). The N-Terminal region of the *Oenococcus oeni* bacteriophage fOg44 lysin behaves as a bona fide signal peptide in *Escherichia coli* and as a *Cis* -inhibitory element, preventing lytic activity on *Oenococcal* cells. J. Bacteriol..

[37] Blankenship B.G., Heffron J.D., Popham D.L. (2015). Lytic enzyme-assisted germination of *Bacillus anthracis* and *Bacillus subtilis* spores. J. Appl. Microbiol..

[38] Lobell R.B., Schleif R.F. (1990). DNA looping and unlooping by araC protein. Science.

[39] Cheng M., Zhang Y., Li X., Liang J., Hu L., Gong P., Zhang L., Cai R., Zhang H., Ge J. (2017). Endolysin lysEF-P10 shows potential as an alternative treatment strategy for multidrug-resistant *Enterococcus faecalis* infections. Sci. Rep..

[40] Zhou Y., Zhang H., Bao H., Wang X., Wang R. (2017). The lytic activity of recombinant phage lysin lysKΔamidase against staphylococcal strains associated with bovine and human infections in the Jiangsu province of China. Res. Vet. Sci..

[41] Kaur J., Singh P., Sharma D., Harjai K., Chhibber S. (2020). A potent enzybiotic against methicillin-resistant *Staphylococcus aureus*. Virus Genes.

[42] Suo Y., Hardy S.J.S., Randall L.L. (2015). The basis of asymmetry in the secA:secB complex. J. Mol. Biol..

[43] Lai M.-J., Lin N.-T., Hu A., Soo P.-C., Chen L.-K., Chen L.-H., Chang K.-C. (2011). Antibacterial activity of *Acinetobacter baumannii* phage ϕAB2 endolysin (LysAB2) against both gram-positive and gram-negative bacteria. Appl. Microbiol. Biotechnol..

[44] Plotka M., Szadkowska M., Håkansson M., Kovačič R., Al-Karadaghi S., Walse B., Werbowy O., Kaczorowska A.-K., Kaczorowski T. (2020). Molecular characterization of a novel lytic enzyme lysC from *Clostridium intestinale* URNW and its antibacterial activity mediated by positively charged N-terminal extension. Int. J. Mol. Sci..

[45] Osipovitch D.C., Therrien S., Griswold K.E. (2015). Discovery of novel *S. Aureus* autolysins and molecular engineering to enhance bacteriolytic activity. Appl. Microbiol. Biotechnol..

